# Downregulation of PITX2 inhibits the proliferation and migration of liver cancer cells and induces cell apoptosis

**DOI:** 10.1515/biol-2021-0133

**Published:** 2021-12-28

**Authors:** Kebinuer Tuerxun, Shufang Zhang, Yuexin Zhang

**Affiliations:** Department of Infection and Liver Disease Center, The First Affiliated Hospital of Xinjiang Medical University, No. 137, Liyushan South Road, Urumqi, Xinjiang, 830054, China

**Keywords:** PITX2, liver cancer, proliferation, metastasis

## Abstract

Paired-like homeodomain 2 (PITX2) functions as a transcription factor to participate in vertebrate embryogenesis, and dysregulated PITX2 expression was associated with the progression of various cancers. The functional role of PITX2 in tumorigenesis of liver cancer remains unknown. Western blot analysis showed that expression levels of PITX2 were enhanced in the liver cancer tissues and cells. siRNAs targeting *PITX2* induced downregulation of PITX2 in liver cancer cells. siRNA-induced knockdown of *PITX2* decreased liver cancer cell viability and proliferation, while promoting cell apoptosis by increasing cleaved-PARP, cleaved caspase 3, and cleaved caspase 9. The knockdown of PITX2 repressed liver cancer cell migration and invasion. In conclusion, elevated PITX2 expression was associated with liver cancer progression through repression of cell apoptosis and promoting cell proliferation and metastasis, and silencing of *PITX2* might serve as a potential therapeutic strategy for the treatment of liver cancer.

## 1 Introduction

Liver cancer is the fifth most common cancer globally and the third leading cause of cancer-related mortality with increasing incidence and occurrence [1]. Alcohol, hepatitis B virus, or hepatitis C virus often induce cirrhosis and result in liver cancer, and antiviral therapy reduces the risk of liver cancer [2]. For example, medical herbs that reduced oxidative stress, suppressed inflammatory response, induced apoptosis, and protected hepatocytes from cirrhosis [3] were widely used in the treatment of liver cancer [4]. However, despite recent advances in the diagnosis and treatment of liver cancer, the long-term prognosis for the patients remains poor [5]. For patients with advanced liver cancer, the overall 5-year survival is only 5%, mainly due to intrahepatic recurrence and metastasis [6]. Therefore, exploring the molecular mechanism of liver cancer progression and metastasis is of clinical significance and would improve the prognosis of the patients.

Paired-like homeodomain 2 (PITX2), which plays an important role in cell proliferation and differentiation, is involved in the development of eyes, teeth, and abdominal organs [7]. Increasing evidence has shown the key role of PITX2 in tumorigenesis. For example, enforced PITX2 expression in ovarian cancer cells enhanced proteins involved in cell cycle regulation, such as Cyclin D1 and c-myc, to promote cell proliferation and growth [8]. PITX2 transcriptionally activated ATP binding cassette subfamily B member 1, a multidrug transporter, to protect against doxorubicin toxicity in renal cancer cell lines [9]. PITX2 also transcriptionally regulated interferon-inducible transmembrane protein 1 to promote the letrozole resistance in breast cancer cells [10]. In addition to the tumor promotive role, PITX2 also functioned as a poor prognostic biomarker for breast cancer [11], colorectal carcinoma [12], head and neck squamous cell carcinoma [13], and esophageal squamous cell carcinoma [14]. However, the functional role and mechanism of PITX2 in liver cancer have not been reported yet.

This study first aimed to investigate the expression levels of PITX2 in liver cancer tissues and cells. The effects of PITX2 on the proliferation, migration, invasion, and apoptosis of liver cancer cells were then assessed. The investigation of the relevant mechanisms involved in PITX2-mediated liver cancer progression provided a theoretical basis for the search and development of new targets for the diagnosis and treatment of liver cancer.

## 2 Materials and methods

### 2.1 Tissue samples

Forty pairs of liver cancer tissues and the adjacent normal liver tissues were acquired from patients recruited in the First Affiliated Hospital of Xinjiang Medical University. The clinicopathological data for patients are shown in Table 1.

**Table 1:** Correlation/association between PITX2 expression and clinicopathological features

		PITX2		Fisher/*X* ^2^	*p*
Clinical features		High (*n* = 20)	Low (*n* = 20)		
Age	>60	8	6	0.440	0.507
	≤60	12	14		
Gender	Male	14	13	0.114	0.736
	Female	6	7		
Tumor size	>5 cm	6	5	0.125	0.723
	≤5 cm	14	15		
Clinical stages	I–II	4	11	5.227	0.022
	III–IV	16	9		
Metastasis status	Yes	13	5	6.465	0.011
	No	7	15		


**Informed consent:** Informed consent has been obtained from all individuals included in this study.
**Ethical approval:** The research related to human use has been complied with all the relevant national regulations, institutional policies and in accordance with the tenets of the Helsinki Declaration, and has been approved by the Ethics Committee of the First Affiliated Hospital of Xinjiang Medical University.

### 2.2 Cell culture and transfection

Liver cancer cells (Hep3B, MHCC-97H, Huh7, and HCCLM3) and human fetal hepatocyte line (L-02) were purchased from the ATCC (Manassas, VA, USA). Cells were grown in DMEM medium (Gibco BRL, Gaithersburg, MD, USA) containing 10% fetal bovine serum (Gibco BRL).

siRNA targeting *PITX2* (si-PITX2-1#: 5′-CAGCCUGAAUAACUUGAACT T-3′ and si-PITX2-2#: 5′-GCCGACTCCTCCGTATGTTTA-3′) and the negative control (si-NC 5′-UUCUCCGAACGUGUCACGUTT-3′) were synthesized in RiboBio (Guangzhou, China). Huh7 was plated in a 96-well plate and transfected with 20 nM siRNAs by Lipofectamine 2000 (Thermo Fisher Scientific, Waltham, MA, USA), and the cells were used for functional assays 2 days post-transfection according to previous research [15].

### 2.3 Cell viability and proliferation

Huh7 (1 × 10^3^ cells/well) cells were plated in a 96-well plate and then indicated transfections were performed. Cells were cultured in the plate for 1, 2, 3, or 4 days. A total of 0.5 mg/mL MTT solution (Sigma-Aldrich, St. Louis, MO, USA) was added to each well and incubated with the cells for 3 h. Lysis buffer (10% SDS in 0.01 M HCl) was added, and the absorbance at 450 nm in each well was measured by Spectrometer (Thermo Fisher Scientific). Huh7 (1 × 10^3^ cells/well) cells were plated in a 6-well plate and then performed with indicated transfections. Cells were cultured for 2 weeks. Fixed cells were stained with 0.4% crystal violet (Sigma-Aldrich), and the colonies were measured under a light microscope (Olympus Corp. Tokyo, Japan) according to previous research [15].

### 2.4 Cell apoptosis

Huh7 was plated in a 6-well plate and then performed with indicated transfections. Cells (1 × 10^6^ cells) were treated with trypsin and resuspended in 100 µL of Annexin V‑binding buffer (Thermo Fisher Scientific) and then incubated with 5 µL of Annexin V-FITC (Thermo Fisher Scientific) for 15 min. A total of 400 µL of Annexin V-binding buffer containing 2 µL of propidium iodide solution (2 mg/mL) was used to incubate the cells. Attune™ Flow Cytometer (Thermo Fisher Scientific) was used to detect the cell apoptosis rate according to previous research [16].

### 2.5 Cell migration and invasion

Huh7 (5 × 10^3^ cells/well) was plated in a 6-well plate and then performed with indicated transfections. Scratch wounds were generated in the monolayer of cells in each well via a pipette tip. The wounds were calculated under the light microscope (Olympus) 24 h later. Huh7 with indicated transfections (1 × 10^5^ cells) in 200 μL of serum-free DMEM medium were plated in the upper chamber of Matrigel-coated well (24-well, Corning, Tewksbury, MA, USA). A total of 400 µL of DMEM containing 10% fetal bovine serum was added to the lower chamber. Cells in the lower chamber were stained with 1% crystal violet 24 h later before being counted under the microscope (Olympus), according to previous research [17].

### 2.6 Western blot

Proteins were extracted from liver cancer tissues or cells via RIPA Lysis and Extraction Buffer (Thermo Fisher Scientific), and the protein concentrations were assessed by an acid protein kit (Thermo Fisher Scientific). Proteins were separated by sodium dodecyl sulfate-polyacrylamide gel and electro-transferred onto PVDF membrane (Millipore, Bedford, MA, USA) before blocking with 5% BSA. The membranes were then incubated overnight with primary antibodies: anti-PITX2, anti-cleaved-PARP (1:2,000, Cell Signaling, Beverly, MA, USA), cleaved caspase 3/9, and anti-cleaved caspase 3/9 (1:2,500, Cell Signaling), and anti-GAPDH (1:4,000, Cell Signaling). The membranes were incubated with corresponding horseradish peroxidase-labeled secondary antibody (1:5,000; Cell Signaling) before detection of the immunoreactivities of the bands in the membranes via enhanced chemiluminescence (KeyGen, Nanjing, China) according to previous research [16].

### 2.7 Statistical analysis

Data were expressed as mean value ± SEM, and performed with one-way analysis of variance or student’s *t* test under GraphPad Prism software. The *p* value < 0.05 was considered as statistically significant.

## 3 Results

### 3.1 Elevated PITX2 in liver cancer

**Figure 1:**
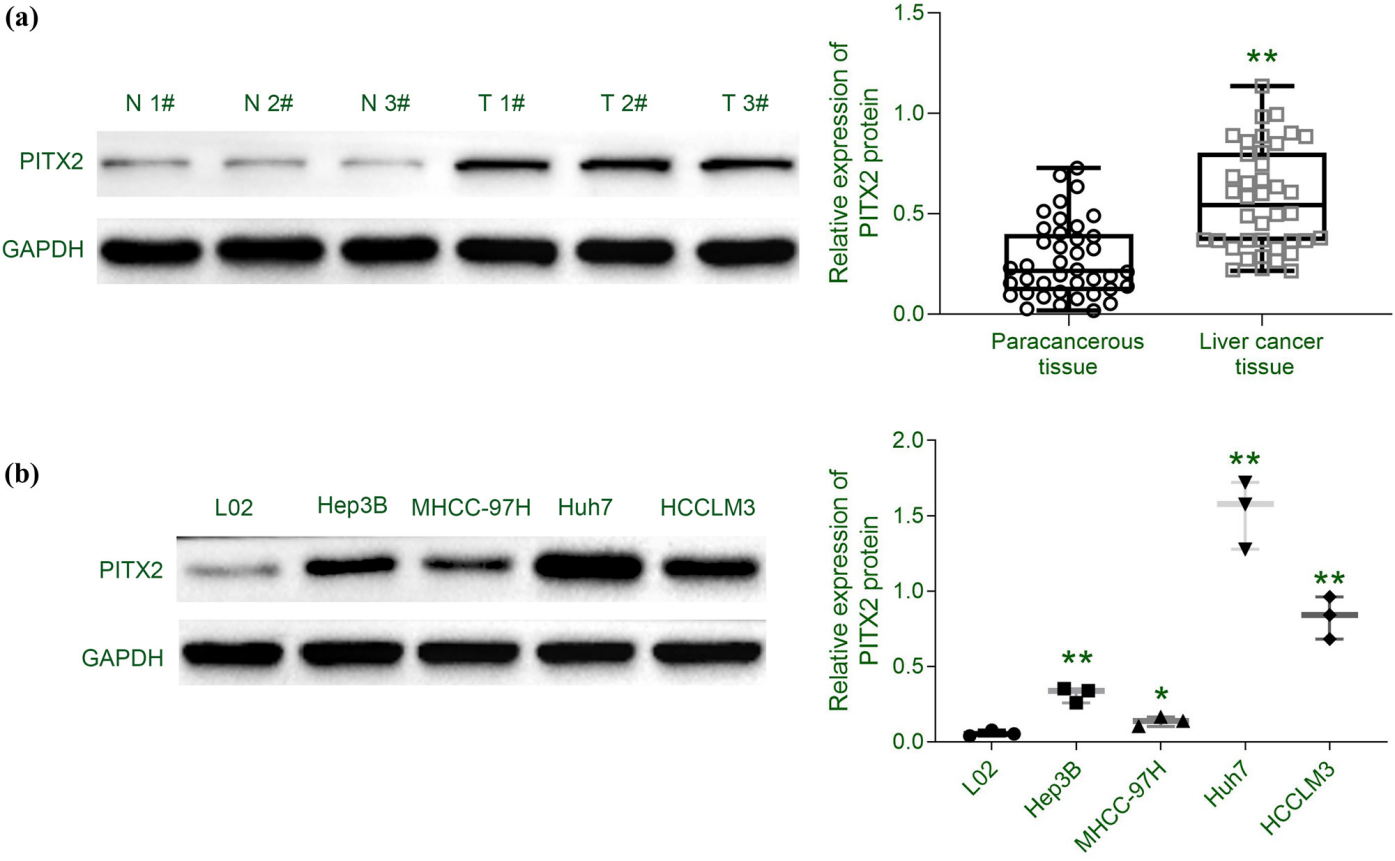
Elevated PITX2 in liver cancer. (a) Protein expression of PITX2 was elevated in liver cancer tissues compared to the adjacent liver tissues. (b) Protein expression of PITX2 was elevated in liver cancer cells (Hep3B, MHCC-97H, Huh7, and HCCLM3) compared to human fetal hepatocyte line (L-02). *N* = 3. **p < 0.01.

A total of 40 pairs of liver cancer and the adjacent liver tissues were collected and applied for western blot analysis to determine the expression level of PITX2 in liver cancer. The result showed that PITX2 was elevated in the liver cancer tissues compared with the adjacent normal tissues (Figure 1a). Analysis of the relationship between PITX2 expression and clinicopathological features of the patients showed that high PITX2 expression was significantly associated with the patients’ clinical stages and metastasis status (Table 1). Moreover, liver cancer cells (Hep3B, MHCC-97H, Huh7, and HCCLM3) showed increased expression of PITX2 compared with the human fetal hepatocyte line (L-02) (Figure 1b), suggesting that elevated PITX2 might be involved in liver cancer progression.

### 3.2 Silence of PITX2 repressed liver cancer cell proliferation

**Figure 2:**
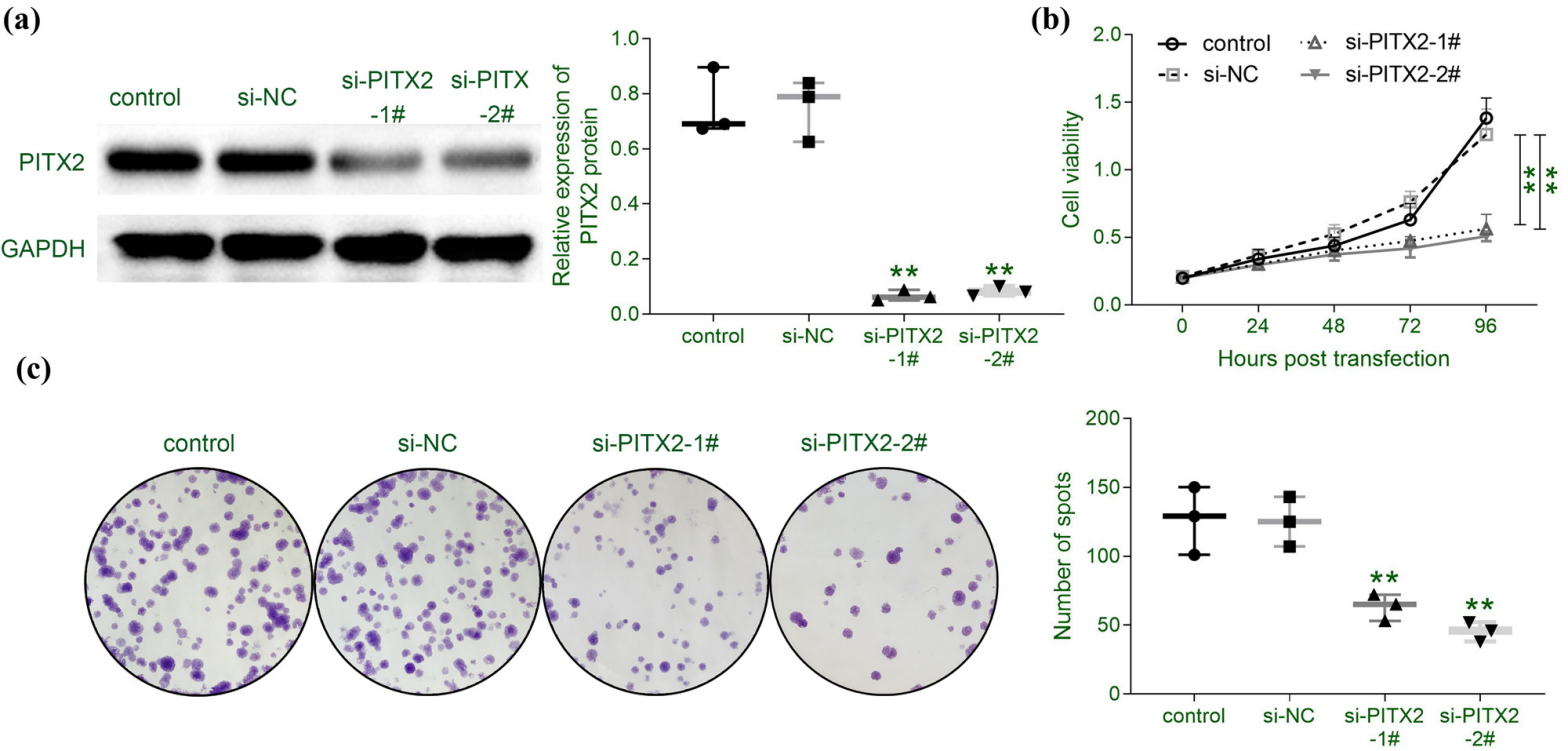
Silence of PITX2 repressed liver cancer cell proliferation. (a) Transfection with siPITX2 decreased protein expression of PITX2 in Huh7. *N* = 3. (b) Transfection with siPITX2 decreased cell viability of Huh7. *N* = 3. (c) Transfection with siPITX2 decreased cell proliferation of Huh7. *N* = 3. *p < 0.05, **p < 0.01.

**Figure A1:**
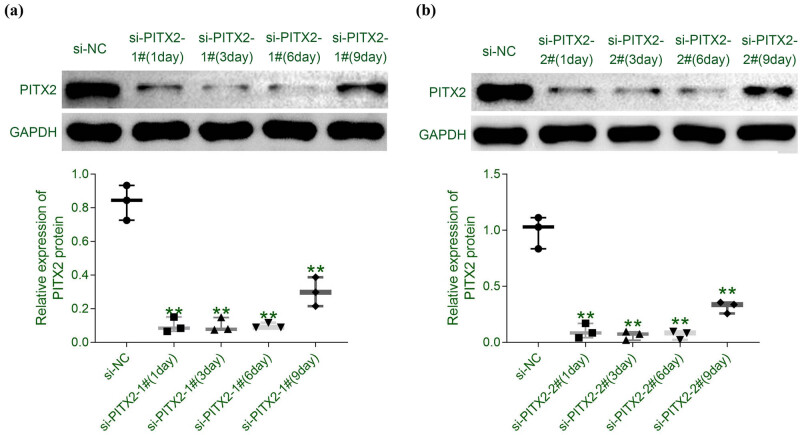
The stability of si-PITX2-#1 (a) and si-PITX2-#2 (b) transfection in Huh7. *N* = 3. **p < 0.01.

A loss of function assay through siRNA-mediated knockdown of PITX2 was applied to investigate the effect of PITX2 on liver cancer cell proliferation. Transfection with siPITX2 in Huh7 showed lower protein expression of PITX2 than the control cells or cells transfected with siNC (Figure 2a). The stability of the PITX2 knockdown is shown in Appendix Figure A1. Huh7 cells transfected with siPITX2 showed lower cell viability than the control or cells transfected with siNC (Figure 2b). Moreover, cell proliferation of Huh7 was repressed by si*PITX2* transfection as demonstrated by lower colony numbers in cells transfected with siPITX2 than cells transfected with siNC or control (Figure 2c). These results demonstrated the anti-proliferative role of PITX2 silencing on liver cancer cells.

### 3.3 Silence of PITX2 repressed liver cancer cell migration and invasion

**Figure 3:**
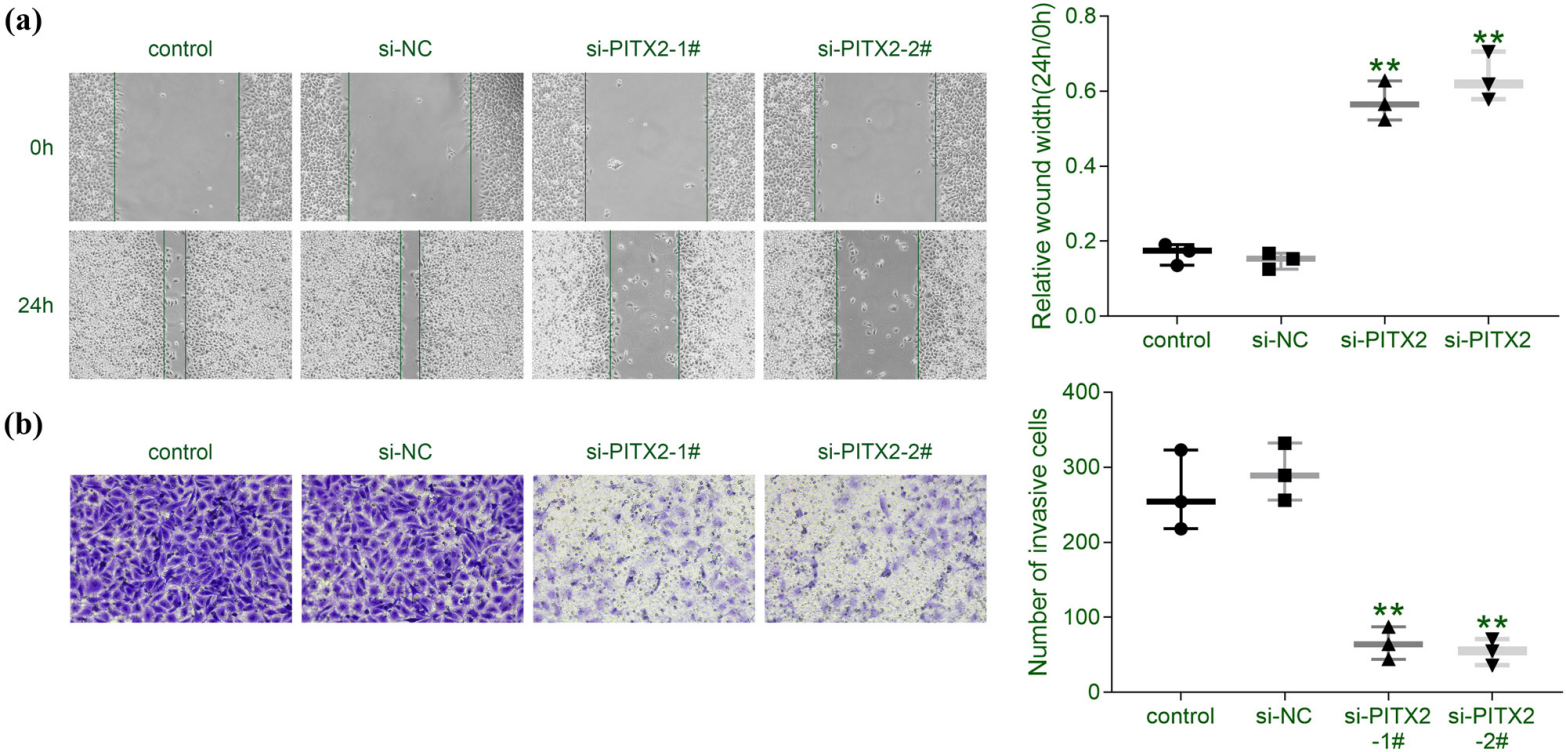
Silence of PITX2 repressed liver cancer cell migration and invasion. (a) Transfection with siPITX2 decreased cell migration of Huh7. *N* = 3. (b) Transfection with siPITX2 decreased cell invasion of Huh7. *N* = 3. **p < 0.01.

The effect of PITX2 on liver cancer cell invasion was then assessed by wound healing and transwell assays. Results revealed that siRNA-mediated knockdown of PITX2 repressed cell migration of Huh7 (Figure 3a). Moreover, the cell invasion of Huh7 was also retarded by the knockdown of *PITX2* (Figure 3b), suggesting the anti-invasive effect of PITX2 silencing on liver cancer cells.

### 3.4 Silence of PITX2-promoted liver cancer cell apoptosis

**Figure 4:**
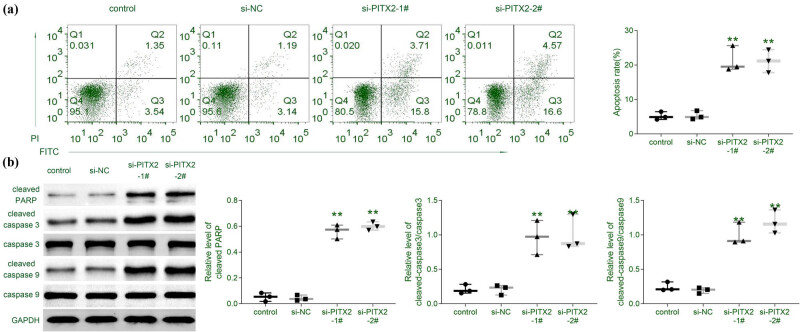
Silence of PITX2 promoted liver cancer cell apoptosis. (a) Transfection with siPITX2 promoted cell apoptosis of Huh7. *N* = 3. (b) Transfection with siPITX2 increased protein expression of cleaved-PARP, cleaved caspase 3, and cleaved caspase 9 in Huh7. *N* = 3. *p < 0.05, **p < 0.01.

In addition to the anti-proliferative and anti-invasive effects of PITX2 silence on liver cancer cells, the effect of PITX2 on liver cancer cell apoptosis was also assessed by flow cytometry and western blot assays. The result indicated that siRNA-mediated knockdown of *PITX2* promoted the cell apoptosis of Huh7 (Figure 4a). Moreover, protein expression of cleaved-PARP, cleaved caspase 3, and cleaved caspase 9 in Huh7 were increased by knockdown of *PITX2* (Figure 4b), suggesting the anti-apoptotic effect of PITX2 on liver cancer cells.

## 4 Discussion

Transcription factors encoded by homeobox genes are implicated in cell differentiation and participate in embryonic development [18]. The association between homeobox transcription factors and tumorigenesis has been widely investigated in recent research [18]. Hlx, Hex, and prospero-related homeobox 1 genes were reported to be involved in liver bud development [19]. Prospero-related homeobox 1 has been shown to repress hepatocellular carcinoma cell proliferation [20]. Bicoid-related PITX genes are shown to be responsible for cell survival [21], and PITX1 functioned as a tumor suppressor in hepatocarcinogenesis [22]. Since PITX2 has been reported to be involved in tumorigenesis of various tumors, the involvement of PITX2 in liver cancer was investigated in this study.

First, our results showed that PITX2 was elevated in the liver cancer tissues and cells. The diagnostic or prognostic roles of PITX2 in breast cancer [11], colorectal carcinoma [12], head and neck squamous cell carcinoma [13], and esophageal squamous cell carcinoma [14] have been reported before. The association between PITX2 expression and clinicopathological features of patients with liver cancer should be investigated in further research to explore its diagnostic or prognostic roles in liver cancer.

Second, the functional assays in this study identified the promoting role of PITX2 in liver cancer progression. Silencing of *PITX2* reduced cell viability of liver cancer cells, repressed cell proliferation, migration, and invasion, and promoted cell apoptosis. Almost 80% of PITX2 expression was reduced by transfection with si-PITX2-1# or 2#, suggesting that other homeobox transcriptional factors might also be implicated in the pathogenesis of liver cancer. Moreover, the epithelial-mesenchymal transition has been shown to be involved in liver cancer invasion and metastasis [23], and over-expression of *PITX2* contributed to the gain of mesenchymal markers and loss of epithelial markers to promote epithelial-mesenchymal transition of ovarian cancer [24]. The effect of PITX2 on epithelial-mesenchymal transition of liver cancer should be investigated to confirm the promoting role of PITX2 on liver cancer metastasis.

Aberrant activation of Wnt/β-catenin participates in tumor cell proliferation, migration, invasion, differentiation, and apoptosis [25], and inhibitors of the Wnt/β-catenin pathway have been applied in clinical trials for drug therapy of tumors [25]. Alcohol, hepatitis B virus, or hepatitis C virus could induce activation of Wnt/β-catenin in hepatic precancerous lesions and cancerous foci [26]. Inhibitors targeting Wnt/β-catenin signaling have been preclinically and clinically evaluated in liver cancers [27]. *PITX2* was reported to be a Wnt signaling target gene and contributed to metastatic prostate cancer [28]. Moreover, *PITX2* has been shown to interact with Wnt genes involved in canonical, noncanonical, or other pathways, such as WNT2/5A/9A/6/2B, to promote ovarian adenocarcinoma cell proliferation [29]. Activation of the Wnt/β-catenin signaling pathway by PITX2 contributed to lung adenocarcinoma progression [30]. Therefore, PITX2 might contribute to liver cancer progression through activation of the Wnt/β-catenin signaling pathway. However, the *in vivo* animal model should be used to assess the effects of PITX2 on liver cancer cell growth.

In summary, this study demonstrated the protective effect of PITX2 silencing on liver cancer cell proliferation, migration, and invasion. This study might provide a novel therapeutic target for the treatment of liver cancer.
